# New Appliances and Things Medical

**Published:** 1904-10-01

**Authors:** 


### NEW APPLIANCES AND THINGS MEDICAL.

[We shall be glad to receive, at our Office, 28 & 29 Southampton Street, Strand, London, W.C., from the manufacturers, specimens of all new preparations and appliances which may be brought out from time to time.]

### CHLOROFORM.

(The United Alkali Co., Gaskell Deacon Works, Widnes.)

We have chemically examined the chloroform before us and find that it perfectly fulfils the requirements of the British Pharmacopœia. Its specific gravity is 1·4924, showing that it contains, according to the Pharmacopœial regulations, a small quantity of alcohol. It is neutral to litmus, and evaporates without leaving any foreign odour. It contains no free chlorine, and no chlorides, and no decomposition products. It follows from this analysis that the chloroform is pure and can be thoroughly recommended for anæsthetic purposes.

In addition to its freedom from impurities, which of course is an essential qualification of chloroform used for the production of anæsthesia, the sample under consideration possesses another strong claim to recommendation, for we understand that it is placed on the market at a price which compares very favourably with the cost of other brands in general use.

